# Screening for Familial Hypercholesterolemia in Childhood: An Overview of Current Practices Around the World

**DOI:** 10.3390/children12101364

**Published:** 2025-10-09

**Authors:** Maria Elena Capra, Roberta Sodero, Elisa Travaglia, Giuseppe Banderali, Giacomo Biasucci, Cristina Pederiva

**Affiliations:** 1 Pediatrics and Neonatology Unit, Guglielmo da Saliceto Hospital, 29121 Piacenza, Italy; m.capra@ausl.pc.it; 2 Pediatrics Unit, Clinical Service for Dyslipidemias, Study and Prevention of Atherosclerosis in Childhood, ASST-Santi Paolo e Carlo, 20142 Milan, Italycristina.pederiva@asst-santipaolocarlo.it (C.P.); 3 Department of Medicine and Surgery, University of Parma, 43125 Parma, Italy

**Keywords:** familial hypercholesterolemia, children, pediatric, screening, worldwide

## Abstract

Familial hypercholesterolemia (FH) is a common genetic disorder with a fairly constant worldwide prevalence of 1 case per 311 individuals worldwide. It is characterized by severe hypercholesterolemia from birth, early atherosclerosis and death from cardiovascular disease at a young age. Diagnosis and treatment from childhood are essential to reduce cardiovascular mortality. Many countries have developed a strategy of implementing pediatric screening, which has led to an increase in diagnoses. This paper evaluates the screening strategies implemented in different countries worldwide. First, we examined which schemes were preferred in various national contexts in Europe. Next, we evaluated the screening methods used in the US, Canada, Australia and Japan. Finally, we researched the screening strategies proposed in some low-resource countries, discovering the difficulties and limitations they face. We have highlighted a wide range of realities, from small-scale pilot studies to cutting-edge proposals. We have also emphasized that, while the topic is certainly of interest, it is burdened by multiple difficulties and unresolved questions.

## 1. Introduction

Familial hypercholesterolemia (FH) is one of the highest prevalence monogenic diseases in the general population, with an estimated 1 case per 311 individuals for the heterozygous form (HeFH) and about 1 in 300,000 for the homozygous form (HoFH) [1]. It is an autosomal dominant disorder with complete penetrance, caused by an alteration in cholesterol metabolism that leads to a deficiency in the hepatic removal of low-density lipoprotein (LDL), resulting in the plasma accumulation of LDL-cholesterol (LDL-C) from birth [2].

This condition exposes affected individuals to persistently high levels of LDL-C, with progressive lipid deposition at the vascular endothelium and acceleration of the atherosclerotic process. Histopathological findings such as those documented in the Fate of Early Lesions in Children (FELIC) study, have demonstrated the presence of lipid streaks as early as prenatally in fetuses exposed to maternal hypercholesterolemia, underscoring the early onset of vascular damage [3].

Even in pediatric age, early signs of atherosclerosis are evident in individuals with FH. In particular, in children with an untreated heterozygous form, increased carotid intima-media thickness (c-IMT) by ultrasound examination has been found as early as 8 years of age, compared with unaffected peers [4].

In more severe cases, such as in HoFH, the severity of hypercholesterolemia can lead to severe clinical cardiovascular manifestations as early as the first years of life, including ischemic events or myocardial infarction before the age of 10 years [1]. The timing of clinical onset of the disease varies by genotype: generally, HoFH subjects develop symptoms before age 20, those with the heterozygous form between 20 and 40 years, and in unaffected subjects, cardiovascular disease occurs around the fifth decade of life or earlier in case of other risk factors.

In most cases, FH is determined by pathogenic variants in the *LDLR* gene, which codes for the LDL receptor [2]. In a minority of patients, pathogenic variants in other genes have been identified, including *APOB* (5–10%), which encodes for the LDL receptor ligand protein, and *PCSK9* (1–3%), which regulates LDL receptor degradation. Mutations in the *LDLRAP1* gene, however, are responsible for the recessive form of the disease. In about 5–30% of cases, no specific genetic cause can be identified, suggesting a polygenic basis or the involvement of yet unknown genes [4].

In this paper, we have attempted to provide an overview of the various screening methods for FH implemented in different countries. Starting with Europe, which has the largest number and variety of programs overall, we then looked at the United States and Canada, as well as Australia and Japan. Finally, we aimed to evaluate the situation in low-resource countries, such as those in Africa, Latin America and India, where awareness of the disease is still in its infancy. Although they are united by the intention to identify and treat FH from a young age, these countries are burdened by many unresolved issues and have very different national conditions and realities.

## 2. Materials and Methods

The aim of our review was to analyze screening strategies for FH in pediatric patients implemented in different countries worldwide. The following three steps were taken to conduct this narrative review: conducting the search, looking through abstracts and full texts, and evaluating the findings. The search was performed by two independent researchers and then the results obtained were compared, discussed, and implemented. To accomplish this, articles from 1992 to 2025 were gathered and chosen from the Pub-Med, EMBASE, Scopus, Science Direct, Web of Science, and Google Scholar databases in order to identify relevant studies that aligned with the review’s progression. The most recent literature analysis was conducted in August 2025. The search encompassed double-blind, randomized controlled studies, controlled clinical trials, randomized placebo-controlled trials, and systematic reviews. The following combinations of keywords were used: “familial hypercholesterolemia” AND “children” OR “pediatric” OR “adolescents” AND “screening” AND “Italy” OR “Europe” OR “USA” OR “Japan” OR “Low income Countries” OR “Middle Income Countries”. Only full papers and journals published in English were included in the search. Following a comprehensive search, the abstracts were examined to ensure they were relevant to the topic at hand. After removing all duplicates, the remaining articles’ abstracts were examined to make sure they satisfied the review’s inclusion requirements.

## 3. Familial Hypercholesterolemia Worldwide

### 3.1. FH Prevalence in Distinct Countries

The prevalence of FH reported by authors from different countries varies significantly among geographic areas due to genetic, ethnic, and environmental differences, as well as the uneven presence of screening programs and cultural awareness.

In Europe, the estimated prevalence of heterozygous FH is generally between 1:200 and 1:250. A study conducted in Catalonia on a cohort of more than 2.5 million people identified a prevalence of 1:192 for HeFH and 1:425,774 for HoFH, confirming a high burden of disease even in high-income countries [5]. Despite this, it is estimated that less than 10% of patients are correctly diagnosed and treated [6].

In the United States (US), the prevalence of HeFH in adolescence is 1:267 [7], while in Japan, it is estimated that the prevalence of HeFH in the general population is 1 in 208 [8].

In Asia, data show similar prevalence rates but with an even lower level of diagnosis. In Pakistan, for example, the estimated prevalence in the general population is 1:273, but depending on the diagnostic criteria applied, it can be as high as 1:182, suggesting a strong under-diagnosis especially in young people [9]. Across the Asia–Pacific region, more than 15 million individuals are estimated to have FH, but clinical awareness and the availability of screening programs remain limited [9]. In Middle Eastern countries, the prevalence is significantly higher, partly due to high inbreeding.

The Gulf FH Registry showed a prevalence of up to 1:232 in the general population, a finding that underscores the need for large-scale identification strategies [10]. Inbreeding contributes to the transmission of founder pathogenic variants that increase the frequency of homozygous forms, making FH a major public health disease in the region [11].

In Latin America, data are still sparse and fragmentary. According to the Ibero-American registry, more than 1.2 million people are estimated to have FH in the region, but less than 1 percent are correctly diagnosed [12]. Major barriers include the lack of diagnostic infrastructure and the absence of shared guidelines.

In Africa, the lack of epidemiological data is particularly pronounced [13,14].

In Oceania, including Australia, although information has been collected on samples that are not very large, the prevalence appears to be overlapping with that in Europe [15].

As can be seen from the data, FH thus represents a common but severely underdiagnosed condition worldwide. Despite evidence of its prevalence and associated early cardiovascular risk, FH often remains unrecognized, underscoring the urgency of improving screening, education, and clinical management programs globally.

### 3.2. The Prague Declaration

The Prague Declaration constitutes a document, based on solid scientific evidence, aimed at improving the early recognition and treatment of FH.

Drafted by international experts, this document was first submitted to the Slovenian EU presidency in October 2021 and, in September 2022, approved by the Czech Ministry of Health at a Senate meeting held in Prague [16].

The statement is based on the assumption, already set out in the 1998 WHO Consultation [17], that disorders of lipid metabolism, or more specifically, as will be better demonstrated, LDL cholesterol levels, constitute an independent cardiovascular risk factor. The presence of effective therapy in reducing this risk enhances the need for early diagnosis. The European Commission itself has called screening for FH “the best practice in the prevention of cardiovascular disease” [18]. By means of the six-point list, crucial elements for screening to become a reality were emphasized with the Prague Declaration, including: setting up universal or selective screening programs with a focus on family cascade screening; promoting newborn screening; and encouraging the adoption of coordinated health policies in the context of an equitable approach and sustainability for public society. The document also stresses the need to ensure the adequate training of territorial health personnel and an integrated approach of the latter with the specialist Lipid Clinic.

The Lipid Clinic in turn should play an active role in coordinating the screening program and promoting first-line care services. Screening, in fact, should take place in an outpatient setting during routine visits such as immunizations or health screenings [19].

Finally, once an appropriate program has been drawn up and adapted to each country, outreach works for citizens about the knowledge of FH, the risks associated with it, and consequently the importance of early diagnosis and treatment are essential.

### 3.3. Screening Strategies

The implementation of screening for FH in childhood is still debated; indeed, FH is a genetic disease, easily identifiable and for which there is an effective and well-coded treatment, yet the clinical manifestation in pediatric age is silent or very nuanced and, if we exclude the homozygous form, is certainly less challenging than other rapidly disabling genetic diseases.

The most effective method to perform population screening is still a debated issue in public health, in relation to both possible implementation and associated cost–benefit. Historically, the US was among the first countries to consider pediatric hypercholesterolemia as a factor of increased cardiovascular risk in adulthood [20,21] and in 2011 a document/guideline specifically on the screening and treatment of hypercholesterolemia in children and adolescents was formulated. According to that document, two main types of screening are considered: a universal one to be addressed to all subjects between the ages of 9 and 11 years, repeatable in case of normality between the ages of 17 and 21 years; another one addressed to subjects at risk (selective screening), i.e., with positive family history for premature cardiovascular events or hypercholesterolemia or with family history that cannot be evaluated (adopted children). In the case of selective screening, the recruitment age is reduced to 2 years [22].

In terms of the genetic characteristics of FH, i.e., a disease with autosomal dominant transmission, and, regarding the fact that a de novo mutation of the genes involved is extremely rare, the identification of a carrier subject presupposes that one of the two parents is affected and that there is a 50% chance for siblings to inherit the disease. The above represents the rationale for what is referred to as cascade screening. In the present case, once a subject with a confirmed diagnosis of FH is identified, testing for first- and possibly second-degree relatives should be planned [23,24]. From a practical point of view, in addition to the lipid profile, relatives are tested for the genetic variant identified in the index case: in fact, the presence of the pathogenic variant confirms the diagnosis even in the presence of cholesterol levels just at the upper limits [25]. This approach appears to be clinically effective and sustainable in terms of cost–benefit analysis, for the individual and for public health. In contrast, for the universal screening, the search for the major mutated genes in FH does not appear to meet cost–benefit criteria; therefore, screening tests involve the determination of the lipid profile alone [26,27].

Screening in the neonatal period is not yet globally standardized, but is carried out on an experimental basis. There are still some points that need to be clarified, such as the type of blood sample and whether a genetic test for FH should be performed at this age or not. In some studies, a blood sample from cord blood was used, and in others, the capillary blood of the newborn [28]. Other open questions are as follows: the biochemical marker, currently identified in the combination of LDL and apo-B [29]; the high costs for the integration of genetic analysis; and finally ethical and privacy issues for the family [30]. At the same time, the literature on the identification of FH in the neonatal period appears to be in favor of economic-clinical benefits, so we hope for the implementation of this form of screening in future clinical practice [31].

**Figure 1:**
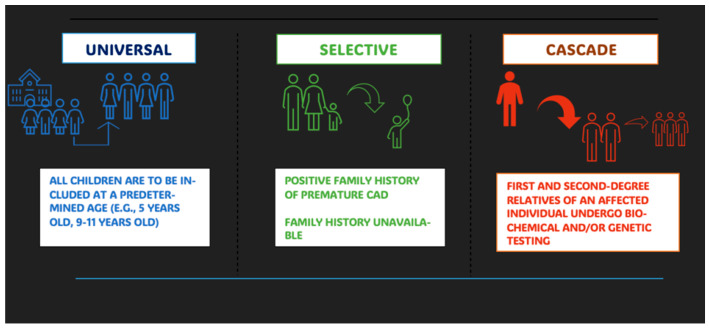
Simplified scheme of FH screening strategies.

The figure below (Figure 1) summarizes the main screening methods.

## 4. Familial Hypercholesterolemia Screening in Europe

Numerous European countries have, over the years, implemented, screening strategies for the identification of FH, which have evolved over time, according to logics of structural adaptation to their respective epidemiological, health and regulatory contexts [18,19].

The introduction of the first systematized national screening program for FH occurred in the Netherlands in 1994, taking the form of a structured “cascade screening” model with government support. This approach involved identification of the index case (proband) by clinical and laboratory criteria (LDL-C ≥ 190 mg/dL/5 mmol/L or presence of tendon xanthomas, early cardiovascular events), followed by molecular genetic testing for diagnostic confirmation. In case of positivity, first-degree family members were sequentially traced and tested and then, according to expanded genealogical criteria, additional family members. The program resulted in the identification of more than 28,000 individuals with FH over a 20-year period, reaching approximately 53% of the expected population based on estimated prevalence, with family participation rates exceeding 90% in the involved households [32,33]. The diagnostic efficiency, coupled with a favorable cost-effectiveness profile, has made this model a benchmark for implementation in other European health care systems [29]. However, the suspension of public funding in 2014 resulted in a significant contraction of new identifications, highlighting the need for structural institutional supports for long-term program sustainability [34].

Following the Dutch experience, Norway and Sweden adopted similar cascade screening programs at the turn of the late 1990s and early 2000s. In Norway, the integration of genetic registries, electronic health records, and the presence of a highly centralized universalistic health care system facilitated the systematic identification of cases and family members, leading to cumulative identification rates of more than 50 percent by 2020 [35]. Sweden has implemented a similar model, with strong integration between primary care levels and specialist centers through the use of clinical–genetic algorithms and coordinated diagnostic pathways [36]. In both contexts, the high degree of computerization and the prevention-oriented health culture favored the stable adoption of the cascade model.

In 1995, Slovenia introduced a completely different paradigm based on universal pediatric screening; this program has been implemented in recent years. All 5-year-old children undergo the determination of total cholesterol during the mandatory pediatric visit under the National Growth Control Program. In the presence of values > 230 mg/dL/>6 mmol/L or >190 mg/dL/>5 mmol/L associated with a positive family history of hypercholesterolemia or early cardiovascular events, the subject is referred to a referral center for in-depth biochemical and targeted genetic testing. The identification of pathogenic variants in the *LDLR*, *APOB*, or *PCSK9* genes results in the initiation of familial cascade screening. Slovenia achieved more than 90% coverage in the targeted pediatric cohort and 57% genetic confirmation rates among individuals with suspected hypercholesterolemia [37]. Screening also enabled the identification of ultra-rare disorders such as lysosomal acid lipase deficiency (LAL-D), confirming the secondary utility of the model in detecting other genetic dyslipidemias. To date, the Slovenian program represents the European benchmark for operational efficiency and systematization of the pediatric diagnostic-therapeutic pathway [37].

Experimental initiatives of ‘universal’ lipid screening integrated with pediatric vaccinations have been launched in Greece, with the aim of exploiting opportunities for routine contact between the child and the health care system for the early identification of FH [38]. The project ran for several years, from 1993 to 2018, and resulted in the identification of a large cohort of children and adolescents [39,40].

In 2017, Austria initiated a selective screening pilot project (FH Kids Austria), which was integrated into school enrollment visits of children aged 5–7 years. Parents were given a standardized questionnaire designed to detect positive family history for dyslipidemia or premature cardiovascular disease. Children with positive results were subjected to plasma lipid testing. In the initial period, when out of more than 66,000 children were evaluated, about 4four percent showed criteria compatible with suspected FH; 20 cases were confirmed among the tested children, who were joined by 17 siblings identified by family screening [41]. Despite the limited size of the cohort tested, the model demonstrated fair identification ability with low cost. However, dependence on the quality and veracity of anamnestic information limits the sensitivity of the method, with potential under-diagnosis estimated at 30 to 50 percent of cases [41].

Germany marked an important methodological advance with the launch of the VRONI (Vorsorgeuntersuchung zur Risikoerkennung von Familiären Hypercholesterinämie) project in 2021. This is a pediatric universal screening program for children between the ages of 5 and 14 years, carried out during U9-U10-U11 preventive examinations (mandatory pediatric preventive examinations performed at approximately 5–7–10 years of age, respectively). The procedure involves measurement of LDL-C from capillary blood; in cases of values ≥ 130 mg/dL/3.4 mmol/L, the subject is referred for genetic testing by targeted sequencing. In cases of molecular confirmation, a “reverse cascade screening” pathway involving testing of the patient’s parents and siblings is activated. The VRONI project has involved more than 28,000 children in Bavaria, identifying pathogenic variants in about 1 percent of tested subjects and elevated lipid values in 7 percent of cases [42].

In Italy, the diagnosis of FH has benefited over the past 10 years from the progressive extension of the LIPIGEN network, a coordinated clinical and laboratory consortium that collects and treats genetically based dyslipidemia [43,44]. The network consists of Specialist Centers for the diagnosis and treatment of familial dyslipidemia, encourages genetic analysis, and has established a large pathology registry for FH [45]. The network also promotes training activities, centralized data collection and the harmonization of diagnostic protocols among different regions. However, there is a lack of uniformity in access to genetic testing, dissemination of clinical suspicion criteria and systematic pediatric care have been highlighted [46].

Spain has also structured a program based on the construction of a national registry promoted by the Fundación Hipercholesterolemia Familiar, with active identification activities and centralized genetic testing, oriented toward cascade screening in family members [47,48]. Cost-effectiveness studies conducted in the country have shown that cascade screening is highly sustainable and is able to induce a significant reduction in preventable cardiovascular events [49]. In Spain, there is also an ongoing project that uses electronic health data to identify electronic red flags that may indicate FH as a second chance to identify possible cases. Over 800 people, including 190 children and adolescents, have been found to have a phenotypic FH profile out of the over 200,000 lipid profiles that were examined. According to a simulation study, this population-level stratification approach might increase the detection rate from the present 14% to 50% and advance the diagnostic timetable by 30 years [50].

In the UK, screening for FH in infancy is a topic of active debate, but a very interesting screening strategy has been implemented: child–parent screening, which uses children as the starting point. The feasibility of this screening method was evaluated over a period of about 2 years on a large population, about 13,000 children, and resulted in the identification of 37 children with genetically confirmed FH, 8 children with clinical FH, and 40 relatives with newly diagnosed FH [51]. In the same years, the UK National Screening Committee, on the other hand, banned universal screening adducing excessive cost as the main reason [52]. However, other studies have demonstrated the economic sustainability of this intervention in the long-term [53]. The UK also has a pathology registry specifically for the pediatric cohort [54].

Finally, data collected by the FHSC Collaboration on more than 48 countries, published recently, indicate a progressive convergence of models toward hybrid strategies, combining opportunistic, cascade screening, and universal pediatric screening [55].

Recommendations from the European Atherosclerosis Society and the European Commission converge on the need to integrate FH screening into preventive public health programs [56], with particular emphasis on pediatric diagnosis and the structuring of genetic networks, national registries, and infrastructure for cross-national interoperability [1].

## 5. Familial Hypercholesterolemia Screening in North America, Australia, and Japan

### 5.1. North America

As mentioned previously, the United States (US) has been working on the diagnosis and treatment of FH starting at pediatric age for several years [20,21]. Universal screening, however, has not been implemented on a large scale in the US context, despite increasingly encouraging data in the literature. One of the oldest studies is the Beaver County Study [57]. That study, one of the longest-running ever conducted, followed patients screened during adolescence (between the ages of 11 and 14) for more than 16 years. The results showed that cholesterol levels measured during adolescence are moderately predictive of those in adulthood, and that early screening can support healthy lifestyle changes (such as improved diet and smoking cessation). Based on these data, the American Academy of Pediatric has proposed universal screening for the pediatric population aged 9 to 11 years and 17 to 21 years [21,22]. The lack of implementation of these guidelines stems from data collected in subsequent years by the US Preventive Services Task Forces (USPSTF). In fact, in 2016 the USPSTF, in a comprehensive systemic review, concluded that the evidence is currently insufficient to recommend universal lipid screening in asymptomatic children and adolescents [58].

Although studies show that elevated pediatric LDL-C levels predict high values in adulthood, robust data on long-term cardiovascular outcomes from early screening and early treatment are lacking [59]. Selective screening, i.e., targeting individuals identified as potentially having FH, has also been proposed alongside universal screening. One of the leading US clinical organizations, the Cleveland Clinic, in 2020 proposed a simple model based on three elements: “detect”, i.e., recognition of symptomatic subjects (cutaneous xanthomas, corneal arch) or those with persistent biochemical changes (LDL > 160 mg/dL). Identification of the affected subject allows pre-cohort treatment (“Treat,” second element) and application of cascade screening (“Ask Family,” third element) for first- and second-degree relatives [60]. In this sense, one could speak of a dual, selective, cascade screening model.

As in Europe [16], there is no rigid, single indication for screening method to be implemented in the United States, however, there is a strong urge to identify and treat FH subjects from an early age [61,62].

### 5.2. Canada

In Canada, the indication for lipid screening was initially directed toward the adult population, particularly for male patients over 40 years of age and female patients over 50 years of age in the absence of cardiovascular risk factors. In presence of CVD risk factors, lipid screening is anticipated. In 2014, with subsequent revision in 2018, Canadian Cardiovascular Society (CCS) guidelines strongly recommended cascade screening (lipid profile and potential genetic testing) among first-degree relatives of patients with FH, with uniform local and national implementation [63]. The same CCS later published an update recommending universal lipid screening for all children within the first decade of life and selective lipid screening for children with a family history of premature cardiovascular events or other risk factors for atherosclerosis starting at age two [64]. Despite the proposed indications, adherence to the different forms of screening is currently very low. Regarding universal screening, a survey conducted in 2019 inferred that only three percent of primary care pediatricians propose lipid profile assessment [65]. The lack of education of general practitioners is associated with poor patient education about FH and the stigma related to genetic testing required for diagnosis. Such issues have been so impactful that they have even compromised adherence to cascade screening [66].

### 5.3. Australia

Australia represents an advanced model approach to FH screening. The most cutting-edge proposal is to offer screening to all children aged 1–2 years, paralleling the vaccination program. The choice of the universal model is based on the high prevalence of FH in indigenous pediatric populations: in a pilot study conducted in Western Australia, out of 448 children screened, 3 were found to have FH, with a prevalence found of 1 in every 150 children [67]. Another study examined the prevalence of FH in a cohort of 17- to 18-year-old adolescents and found a prevalence of 1 in every 267 patients. The same study also compared targeted screening strategies (based on family history) with universal screening, the latter being considered superior in terms of diagnostic coverage [68]. The Australian national program for FH historically involves clinical-genetic cascade screening, which allows identification of family members at risk from an index individual. In a multicenter study also conducted in the Western Australian context, 100 index cases and 366 family members were examined: about 51% of the tested family members had a pathogenic genetic variant compatible with FH. The average number of family members diagnosed per index case was about 2, and treatment led to a reduction in LDL-C of up to 42% in previously untreated subjects [69].

### 5.4. Japan

Japan has fairly recently started to diagnose and treat FH on a large scale [70]. As for universal screening, the guidelines do not give a one-sided indication [71]. However, in Kawaga, one of the most advanced prefectures in the area, a pilot project with universal screening has been active since 2012, offered to all 9- to 10-year-olds in the context of elementary school. In this case, patients are considered positive for screening for LDL cholesterol values > 140 mg/dL/3.6 mmol/L. Once identified, they are then referred to second-level centers where genetic analysis (Next Generation Sequencing) is performed to confirm the diagnosis. Following the latter, cascade screening to family members can then be expected. The goal would be to extend this model to the whole population [72]. In 2022, the Japanese guidelines (JAS 2022) redefined the diagnostic criteria for FH with a significant increase in sensitivity in new diagnoses and established the currently available treatment lines for adults and pediatric patients 10 years of age and older. Associated with these elements was the strong recommendation for screening of first-degree relatives of individuals with FH, a practice whose adoption is very limited with significant interregional variability [73,74].

Another progressive proposal is to include genetic testing in the initial screening phase rather than only using it for diagnostic confirmation. Comparing the application of the guidelines alone with their application combined with genetic analysis has shown that the latter option results in greater diagnostic sensitivity [75].

The screening methods implemented in different countries are summarized in Table 1.

## 6. Familial Hypercholesterolemia Screening in Low-Income Countries

In this chapter, we evaluate the distribution and treatment methods for individuals affected by FH in low-resource countries [80] and investigate whether there are any specific registries or consensus documents. The available literature is very recent and mainly comprises single-center or multicenter studies, as well as treatment documents, which are sometimes produced under the auspices of European Scientific Societies [81,82,83].

### 6.1. Africa

In Africa, the approach to FH, and consequently to screening, is extremely uneven. The principal differences are based on disease awareness and public support, which are highly expressed in Southern Africa (and particularly in South Africa), being defined in Northern Africa, and practically absent in Central Africa [76].

Southern Africa has long been engaged in the study of FH. As early as the 1990s’ an elevated prevalence was found in some ethnic groups residing in the South African territory, particularly in Afrikaners, Jews, and Indians [84]. This highly significant figure (1:80, compared to the global average of 1:250) is explained by the so-called “founder effect” involving the high frequency of certain diseases within closed groups. With this realization, therefore, studies aimed at identifying ethnic-specific gene variants responsible for FH have arisen. Thus, while within founder populations, research has achieved important milestones that can also be exploited in the construction of effective genetic screening; the same cannot be said for the indigenous South African population. In that case, the prevalence has probably been underestimated over the years, precisely because of screening based on pathogenic variants referred to other ethnicities as well as clinical criteria not adapted to the local population [14].

A turning point came in 2016 with the Wits FIND-FH program, organized by the University of the Witwatersrand and aimed at identifying individuals with FH through cascade screening. Based primarily on clinical rather than genetic criteria, it enabled the identification of a large number of individuals, including within the indigenous South African population. Furthermore, 14% of patients identified were under the age of 18 and were unaware of their condition prior to screening. This confirms the usefulness of early screening, particularly in contexts with a high prevalence, such as the one analyzed here [76]. Some recent studies also report data on the treatment of FH in this geographical area [85], and specific documents on diagnosis and treatment are currently being finalized [13].

In North Africa, there are no screening programs and no disease registries for FH. Instead, there are sporadic studies [86] and very recent consensus documents [83,87], resulting in little awareness of the disease and its associated risks. In 2015, a study was conducted on the Middle East and North Africa region (a region defined as MENA) aimed at identifying causative pathogenic variants of FH in this population: only 57 were found in the 17 regions analyzed versus 500 analyzed in countries with active registries. This is associated with a very high incidence of cardiovascular disease. The poor identification of FH in a context with a high rate of consanguinity could be the reason for this oxymoron [11].

### 6.2. Latin America

In Latin America, data on the prevalence and genetics of FH are scarce. A recent systematic review of 30 studies in 10 Latin American countries showed that the estimated prevalence of the heterozygous form (HeFH) is approximately 1 in 500 people, with indications that it may actually be higher (~1:200–300) in some more ethnically mixed populations [88].

Brazil among the various countries is certainly the most advanced in terms of FH knowledge [89]. In 2020, a study conducted on children and adolescents within the Basic Units of the Public Health System (SUS) in Campinas, São Paulo, between 2008 and 2015, aimed at assessing the frequency, type, and severity of dyslipidemia in the pediatric population, was published. A high prevalence of dyslipidemia was also found in this age group, with uneven distribution within the city. Again, in 2018 through the HipercolBrasil program, pathogenic variants were diagnosed in about 50% of index cases and 59% of their family members. The variants identified were mainly in *LDLR* (97%), few in APOB and none in PCSK9 [77,90]. Some studies have also been conducted on the treatment of FH for both heterozygous [91] and homozygous form [92].

Mexican authors have also reported encouraging data. Recently, both a national registry [93] and consensus documents for the diagnosis and treatment of FH have been developed in Mexico [94].

### 6.3. India

As in other low-resource countries analyzed, there is no national registry or centralized database for FH in India. Therefore, data on disease prevalence is very scarce, and the few available studies focus exclusively on populations that have experienced acute cardiovascular events. According to recent work conducted in 2019 in northern India, the prevalence of FH in subjects with coronary artery disease is 15 percent and therefore comparable to what has been described in high-income countries such as Australia [95].

Another critical element is the accessibility of genetic testing, which is offered only in the most urbanized settings. Knowledge regarding the genetic nature of the disease is also low: according to data currently in the literature, up to 37% of cases do not have identified genetic pathogenic variants, 32% can be attributed to LDL variants, 4% to ApoB, and 2% to PCSK9 [96].

Some screening programs for familial hypercholesterolemia (FH) have also been attempted for children, but the context remains one of low awareness and limited resources [78]. In order to build a global FH registry, the EAS-FHSC has collaborated with three Indian groups (in Mumbai, Delhi and Chennai), who are actively contributing to the project [19,79,97].

## 7. Conclusions

This paper provides an overview of the various screening strategies implemented around the world to diagnose FH in children. Following a brief introduction to the disease, including its prevalence and under-diagnosis, we describe the different screening methods (universal, cascade, and selective). We considered the countries that were among the first to suggest large-scale screening. The US was historically one of the first to propose universal screening, starting as early as the 1990s; shortly thereafter, several European countries such as the Netherlands, Sweden, Norway, Greece, and Slovenia implemented national screening programs on extensive samples. We described the situation in Canada, Australia, and Japan, all of which began screening programs in the 2000s. Finally, we examined the situation in low-resource countries, emphasizing that screening programs have only been proposed and implemented in recent years.

As highlighted in two major global studies, FH remains underdiagnosed and undertreated, particularly in children and young people [55,98].

Since the 1990s, the number of countries proposing and implementing screening programs for FH has increased considerably. Many countries have set up specific registries for the disease and produced diagnostic and management documents, starting from pediatric age.

In recent years, the first follow-up studies have been published on patients who were treated as children and have now become young adults. These studies provide evidence of changes in the disease trajectory [99]. However, the follow-up data of children in the long- and very-long-term are currently limited, and, as we have described, the debate on the usefulness and sustainability of diagnosing FH starting from childhood is still active. The Prague Declaration, issued by Europe, supports the need for and the right of children with FH to be diagnosed from childhood onwards. This is supported not only by the scientific community but also by patient associations. However, there are situations in which diagnosing and treating the disease is extremely challenging, mainly due to a lack of knowledge and specialist center, and difficulty accessing treatment.

In conclusion, although there has been significant progress in improving the ability to diagnose FH from childhood in almost every part of the world, several questions remain unanswered. It will be a few years before more solid long-term follow-up data in children are published to definitively support these initiatives.

## Figures and Tables

**Table 1:** Summary of screening strategies in high and low-income countries.

Country	Main Screening Method	Consensus/Guideline Document	Bibliographic References
**Europe**			
**Netherlands (1994)**	National cascade screening since 1994: index case via clinical/lab criteria + genetic testing; family tracing 1st/2nd degree	Dutch Ministry of Health FH Screening Program Guidelines (1994–2014)	Zuurbier et al., 2021 [34]
**Slovenia (1995)**	Universal pediatric screening at age 5 (total cholesterol); genetic confirmation; cascade family screening	National Pediatric Preventive Program—FH Screening Protocol	Groselj et al., 2018 [37];
**Norway (late 1990s)**	Cascade screening integrated with genetic registries and electronic health records; >50% of expected cases identified by 2020	Norwegian Directorate of Health FH Recommendations	Bogsrud et al., 2010 [35]
**Sweden (early 2000s)**	Cascade screening using clinical-genetic algorithms; strong primary-specialist care integration	HEART UK statement adapted for Sweden	Ramaswami et al., 2020 [36]
**Greece (1993–2018, pilot)**	Universal pediatric lipid screening integrated with vaccination visits	Greek Pediatric Lipid Screening Project Protocol	Diakou et al., 2011; Mollaki & Drogari, 2016 [38,39]
**Spain (2004)**	National SAFEHEART registry, cascade clinical-genetic screening; cost-effectiveness proven	SAFEHEART National Registry Protocol	Lázaro et al., 2017 [49]
**Italy (2010s)**	LIPIGEN network: specialist centers, registries, genetic testing; cascade screening	Italian Society of Atherosclerosis—LIPIGEN network	Averna et al., 2017; [44]
**Austria (2017)**	Selective screening (FH Kids Austria) in school entry visits (5–7 y); questionnaire + lipid testing	FH Kids Austria Screening Protocol	Kreissl et al., 2019; [41]
**Germany (2021)**	Universal pediatric screening (VRONI) age 5–14 during preventive visits; LDL-C capillary + genetic testing; reverse cascade	VRONI Pediatric FH Screening Protocol	Sanin et al., 2022 [42,43]
2 **Other countries**			
**USA (AAP rec. 2011)**	Targeted screening + cascade; AAP recommends universal at 9–11 and 17–21 y; USPSTF no recommendation	American Academy of Pediatrics & USPSTF Positions	Shah et al., 2020; Lozano et al., 2016 [58,60]
**United Kingdom (2016)**	Child–parent screening: universal cholesterol testing in toddlers (1–2 y) during vaccination; if positive, genetic testing and parental cascade	UK Child–parent Screening Program (pilot, NHS-supported)	Wald et al., NEJM 2016 [51]
**Australia (2015–2016 pilot)**	Proposed universal pediatric screening at 1–2 years with vaccination; cascade clinical-genetic screening; WA pilot	Australian FH Screening Consensus Statement	Martin et al., 2022; Bell et al., 2015 [67,69]
**Japan (Kagawa pilot 2012; JAS 2022)**	JAS 2022: updated criteria, recommends family screening; Kagawa pilot universal screening at 9–10 y	Japan Atherosclerosis Society (JAS) 2022 Guidelines	JAS 2022; Matsunaga 2025 [73,74,75]
**Canada (2014; update 2018, 2022)**	CCS 2018 and 2022: cascade screening for 1st-degree relatives; universal pediatric screening recommended but low adherence	Canadian Cardiovascular Society FH Guidelines	Brunham et al., 2018; Khoury et al., 2022 [63,64]
**South Africa (1990s)**	Targeted screening in founder populations + cascade (Wits FIND-FH) based on clinical criteria	Wits FIND-FH Program Protocol	Raal et al., 2020 [76]
**Brazil (2008–2015)**	Cascade screening + centralized genetic testing; high prevalence in children	HipercolBrasil National Screening Guidelines	Silva et al., 2018 [77]
**India (2018 pilot; 2024 guidelines call)**	Cascade screening in children and relatives (pilot studies); no national registry yet, but recent national guidelines stress urgent need for structured FH screening	Indian Dyslipidemia Guidelines (Indian Heart Journal, 2024)	Setia et al., 2018; Sawhney & Gupta, 2024 [78,79]

## Data Availability

Not applicable.

## Abbreviations

The following abbreviations are used in this manuscript:

ApoB	Apolipoprotein B
CVD	Cardiovascular Disease
EAS	European Atherosclerosis Society
FH	Familial Hypercholesterolemia
HDL-C	High-Density Lipoprotein Cholesterol
HoFH	Homozygous Familial Hypercholesterolemia
HeFH	Heterozygous Familial Hypercholesterolemia
LDL-C	Low-Density Lipoprotein Cholesterol
LDL-R	Low-Density Lipoprotein Receptor
LDL-RAP1	Low-Density Lipoprotein Receptor-Related Protein-Associated Protein 1
PCSK9	Proprotein Convertase Subtilisin/Kexin type 9
